# Interleukin-1 receptor associated kinase 2 is a functional downstream regulator of complement factor D that controls mitochondrial fitness in diabetic cardiomyopathy

**DOI:** 10.1186/s40779-023-00506-3

**Published:** 2024-01-03

**Authors:** Stanislovas S. Jankauskas, Fahimeh Varzideh, Pasquale Mone, Urna Kansakar, Francesco Di Lorenzo, Angela Lombardi, Gaetano Santulli

**Affiliations:** 1 Department of Medicine, Fleischer Institute for Diabetes and Metabolism (FIDAM), Einstein-Mount Sinai Diabetes Research Center (ES-DRC), Einstein Institute for Aging Research, Albert Einstein College of Medicine, New York, NY 10461 USA; 2 Department of Molecular Pharmacology, Division of Cardiology, Wilf Family Cardiovascular Research Institute, Albert Einstein College of Medicine, New York, NY 10461 USA

**Keywords:** Adipsin, Complement factor D, Interleukin-1, Interleukin-1 receptor-associated kinase like 2 (Irak2), Opa1, Prohibitin (PHB)

## Background

Diabetic cardiomyopathy is a disorder of the cardiac muscle that affects patients with diabetes. The exact mechanisms underlying diabetic cardiomyopathy are mostly unknown, but several factors have been implicated in the pathogenesis of the disease and its progression towards heart failure, including endothelial dysfunction, autonomic neuropathy, metabolic alterations, oxidative stress, and alterations in ion homeostasis, especially calcium transients [1]. In *Military Medical Research*, Jiang et al. [2] sought to determine the functional role of complement factor D (Adipsin) in the pathophysiology of diabetic cardiomyopathy.

## Complement factor D (Adipsin)

Complement factor D is a protein secreted into the bloodstream mainly by adipocytes. It is also known as Adipsin, C3 pro-activator convertase, or properdin factor D esterase. The protein is a member of the trypsin family of serine proteases and has a high level of expression in fat, implying a functional role for adipose tissue in immune system biology. Complement factor D is involved in the alternative pathway of the complement system where it cleaves factor B1 [3].

## High fat diet (HFD) as a model of diabetes

HFD feeding is usually used to obtain animal models of type 2 diabetes mellitus (T2DM), because chronic HFD feeding is capable of inducing hyperglycemia, insulin resistance and glucose intolerance, and similar manifestations of T2DM. Animal models that can nicely recapitulate human T2DM are crucial to examine the pathogenesis and intervention strategies for diabetes and diabetic complications [4, 5].

In their experimental setting, Jiang et al. [2] observed that HFD feeding for 6 months induced a pronounced hyperglycemia as well as diastolic and systolic cardiac dysfunction. They detected reduced serum levels of complement factor D starting at the 2nd month of HFD feeding, which is consistent with previous observations showing that circulating levels of complement factor D decreased in obese patients [6]; such reduction may be due to high activity or resistance, albeit the exact causes are not fully known.

Mass spectrometry (MS) analysis was used to screen the potential proteins that directly interact with complement factor D in cardiomyocytes [2]. The top 5 proteins with high MS scores were interleukin-1 receptor-associated kinase like 2 (Irak2), hemoglobin subunit beta-1 (Hbb-b1), hemoglobin subunit alpha (Hb-α), myosin regulatory light chain 2 (Myl2), and myosin light chain 3 (Myl3). After reviewing the functions of these proteins, Jiang et al. [2] noticed that Irak2 is known to participate in the regulation of cardiomyocyte apoptosis in models of diabetic cardiomyopathy. Additionally, mitochondrial translocation of Irak2 regulates oxidative metabolism in adipocytes [7].

## Irak2

Irak2 is one of the two putative serine/threonine kinases that are associated with the IL-1 receptor upon stimulation. It is involved in the IL-1 receptor/Toll-like receptor (TLR) signaling cascade and is known to act as an adaptor in the TLR-MyD88-TNF receptor associated factor 6 (TRAF6) complex, enabling the downstream activation of NF-κB and thereby regulating inflammation [7]. Irak2 has been shown to translocate in the mitochondrion where it localizes to the inner mitochondrial membrane [7]. At this level, it interacts with prohibitin (PHB), causing PHB to recruit optic atrophy protein 1 (Opa1, also known as dynamin-like 120 kD protein, a fundamental orchestrator of mitochondrial fusion [5]) from the cristae junctions, and suppresses respiratory super-complex formation, ultimately triggering a destabilization of mitochondrial integrity [7].

On these grounds, Jiang et al. [2] further evaluated the interaction between complement factor D and Irak2. Intriguingly, glutathione-S-transferase (GST)-pulldown technique, co-immunoprecipitation, and immunofluorescence co-localization studies established that Irak2 serves as a downstream regulator of complement factor D. Mechanistically, adipose tissue-specific overexpression of complement factor D significantly improved cardiac function and alleviated cardiac remodeling in diabetic cardiomyopathy, but these effects were not observed after *Irak2* knockdown.

**Fig. 1:**
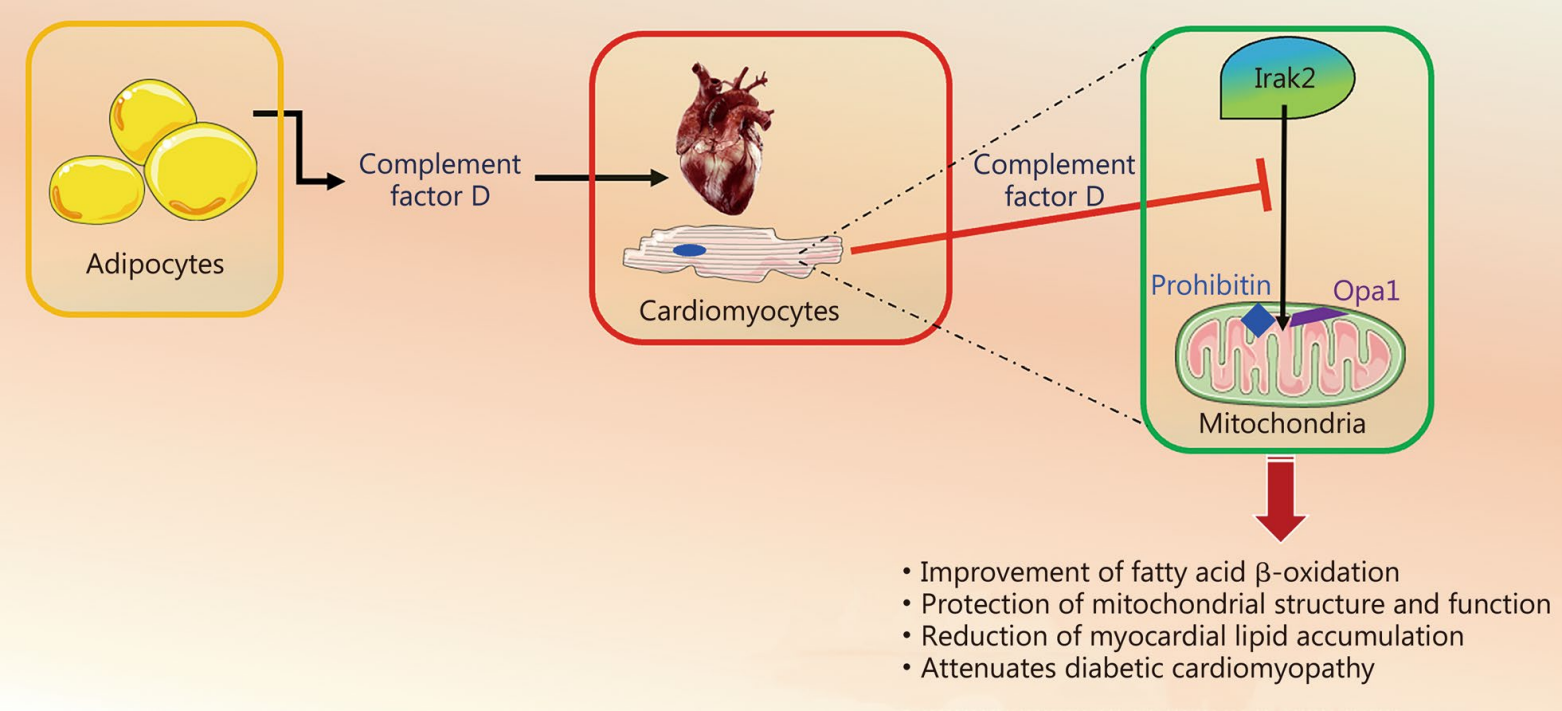
In diabetic cardiomyopathy, interleukin-1 receptor associated kinase 2 (Irak2) translocates in the mitochondrion, where it triggers the recruitment of optic atrophy protein 1 (Opa1, also known as dynamin-like 120 kD protein) from the cristae junctions by prohibitin, destabilizing the organelle integrity and impairing its function. Complement factor D (Adipsin) inhibits the mitochondrial translocation of Irak2, eventually attenuating myocardial dysfunction

The compelling evidence provided in the work led by Jiang et al. [2] indicates that increased complement factor D inhibits Irak2 mitochondrial translocation in the diabetic myocardium, decreasing the interaction between Irak2 and PHB-Opa1, eventually reducing mitochondrial cristae damage and improving mitochondrial fitness (Fig. 1), thereby attenuating the impaired myocardial fatty acid metabolism detected in diabetic cardiomyopathy.

## Strengths and limitations

A strength of the paper is that both immunocolloidal gold electron microscopy and immunoblot analyses confirmed that complement factor D inhibits mitochondrial translocation of Irak2 in diabetic cardiomyopathy, thus reducing the interaction between Irak2 and PHB-Opa1 on mitochondria and improving the structural integrity and function of mitochondria. Limitations include having performed the investigations exclusively in cardiomyocytes, without testing other cardiac cells, and in animal models, without verifying the effects in human cells.

## C57BL/6J vs. C57BL/6N mice

The mice used in this study were C57BL/6J, which are a substrain that is known to carry a mutation in the nicotinamide nucleotide transhydrogenase (*Nnt*) gene, which may affect cellular metabolism. The *Nnt* gene is located on the murine chromosome 13 and encodes a mitochondrial protein involved in mitochondrial metabolism. C57BL/6J mice have a spontaneous in-frame 5-exon deletion in *Nnt* that removes exons 7–11, resulting in inappropriate glucose homeostasis in male C57BL/6J mice [8, 9]. C57BL/6J mice have a normal life span and actually have a robust weight gain and develop obesity and insulin resistance on a HFD. Instead, C57BL/6N lines do not have this mutation and should be preferred in studies investigating mitochondrial phenotypes, and diabetes-related features. Nevertheless, recent investigations suggest that the lack of functional *Nnt* contributes only moderately to the differences in glucose-stimulated insulin secretion and glucose tolerance between the two strains [10].

In summary, serum levels of complement factor D are reduced in HFD-fed mice, associated with hyperglycemia and cardiac dysfunction. Increasing complement factor D inhibits mitochondrial translocation of Irak2, alleviating mitochondrial damage and improving cardiac function in diabetic cardiomyopathy. Irak2 appears crucial in this context, influencing mitochondrial integrity and suggesting a potential therapeutic pathway for diabetic cardiomyopathy.

## Data Availability

Not applicable.

## Abbreviations

HFD: High fat diet
Irak2: Interleukin-1 receptor-associated kinase like 2
Nnt: Nicotinamide nucleotide transhydrogenase
PHB: Prohibitin
T2DM: Type 2 diabetes mellitus
TLR: Toll-like receptor
TRAF6: TNF receptor associated factor 6
